# Should We Rely on the Findings of Each Published Randomized Controlled Study?

**DOI:** 10.5812/hepatmon.8355

**Published:** 2013-03-14

**Authors:** Ali Kabir

**Affiliations:** 1 Department of Epidemiology, Faculty of Public Health, Shahid Beheshti University of Medical Sciences, Center for Educational Research in Medical Sciences, Tehran University of Medical Sciences, Tehran, IR Iran

**Keywords:** Analysis of Variance, Statistics, Nonparametric, Data Interpretation, Statistical

Dear Editor,

Nonalcoholic fatty liver disease (NAFLD) is likely the most common cause of chronic liver disease in many countries with controversies relative to its optimal treatment (1). Despite my interest to the results of Hajiaghamohammadi et al. study (2), on this topic there are shortcomings which should be taken into account before going to their results and implement the findings in clinical practice. First, the multivariate analysis-of-variance (MANOVA) is a generalization of ANOVA allowing multiple dependent variables analysis. Here, multiple dependent variables are considered but not at the same time in the analysis. So, the authors should use ANOVA instead of MANOVA. There is no indication for MANOVA according to the observed results and tables. In addition, even when the basic values are not different (maybe due to low power of the statistical test because of low sample size), it is better to do ANOVA with considering those basic values as covariates. It is due to the fact that there are remained differences which may not be statistically different but exist. Moreover, by considering baseline characteristics, personal differences could have been considered better than the way used in this paper. Additionally, most differences are not significant when there is low sample size. So, non-significant baseline differences can be due to low sample size. There are also some comparisons between subgroups like changes in AST, ALT, FBS, insulin level, and HOMA index in silymarin group in comparison with other groups. Post hoc analysis is needed for such comparisons with three different groups to show which subgroup difference(s) have caused significant difference between three groups at all. They did not mention how they have found these findings neither in methods nor in analysis. According to our post-hoc analysis, many of these differences are not significant despite what the authors have mentioned. Moreover, when we do multiple comparisons, we should use methods like Bonferroni or Holm methods as a correction to adjust the usual P value (3). In such cases, the cut off for rejecting P value is smaller than 0.05 for preventing falsely significant correlations/differences. In other words, type I error (α) is considered more conservative to reject null hypothesis. So, some of the mentioned significant differences might be no longer statistically significant. When we report a randomized controlled trial (RCT) study we can use guidelines like CONSORT (4). Even when we do not follow such checklists, we should mention about crucial issues like blinding, details of inclusion and exclusion criteria, and trial phase of our study. It may be useful to know that many trials in Iran are registered in www.irct.ir from 2008 and receive an Iranian registry clinical trial (IRCT) code which is international and unique. This site is a world health organization (WHO) collaborative center in Iran. It is recommended that journals force their authors to mention such international code (from IRCT or similar sites like clinicaltrial.gov) when they are publishing a clinical trial or an experimental study. It increases the certainty about the quality of that work.

Another important issue is that cut offs are considered according to the normal values, percentiles (quartile), median, and other descriptive statistics. I did not understand why cut off for FBS is 100? There are also references for normal AST and ALT in Iran which is different from 40 IU/L and can be considered for these variables as cut off (5).

I am not sure do these groups meet the criteria of parametric tests completely? According to authors’ claim, distribution of variables was normal. They have not mentioned which approach they have had for determining normality in variables. Even they did not mention the name of the statistical tests used. If, they have only used statistical test, more commonly Kolmogorov-Smirnov (KS), they should be aware that in small sample size (specifically under 30 in each group) this test is not powerful enough to detect difference with normal distribution and may falsely show that the distribution of each variable is normal. In addition, normality should be checked graphically to prevent from such problem and the assessing is normality assumption highly violated? We did not have raw data and were unable to check these assumptions. However, we did Bartlett test showing that differences among standard deviations (SDs) are not significant in all of these comparisons. Equality of variances is more important factor than normality by KS test for searching pre-assumptions of the parametric tests. Variances are equal and it expresses that the data meet criteria for parametric tests in all variables and there is no need to do Kruskal-Wallis test instead of ANOVA. Authors have used parametric tests truly. Interestingly, when we compared mean differences (before and after treatment) between three groups we found that P value of ANOVA is 0.713, 0.277, 0.681, 0.741, 0.109, 0.196, 0.255, and 0.078 for weight, BMI, TG, cholesterol, AST, ALT, insulin level, and HOMA-IR, respectively. Only FBS has significant P value (< 0.0001). So, the results are completely different from what has been mentioned in the paper. They have also compared the results of before and after treatment in each one of the three groups in Table 4 and mentioned which one has more effect. It is advised to use paired t-test in such table to have more accurate conclusion that which one has statistically significant effect on these metabolic and anthropometric variables. There are also some small issues better to be addressed:

1- How they have approached to their missing data? Table 3 shows that there has been missing data.

2- Do all sonographies have been done by one sonographer? If not, what about inter-observer agreement? If yes, what about intra-observer agreement? Evidence shows that the lack of specific and sensitive noninvasive tests for NAFLD limits reliable detection of the disease (1). In such situation, at least we should try to validate our data specifically when the subject itself is at higher risk of low reliability.

3- They have used the phrase “Parameters of participants” in multiple places. We should use the word “parameter” when we are assessing some specifications (like mean and SD) of the target population and not the sample.

4- In the results of the abstract, authors have presented that P < 0.01 for all mentioned variables. However, according to Table 2, P value of reduction in average of cholesterol is 0.027 which is larger than 0.01.

5- They have mentioned that “increased levels of liver enzymes AST and ALT” were among their inclusion criteria. However, there are cases with AST lower than 40 according to Table 1.

6- The unit of FBS seems to be mg/dl and not mmol/L in this study.

## References

[1] Salt WB, 2nd (2004). Nonalcoholic fatty liver disease (NAFLD): a comprehensive review.. J Insur Med..

[2] Hajiaghamohammadi AA, Ziaee A, Oveisi S, Masroor H (2012). Effects of metformin, pioglitazone, and silymarin treatment on non-alcoholic Fatty liver disease: a randomized controlled pilot study.. Hepat Mon..

[3] Aickin M, Gensler H (1996). Adjusting for multiple testing when reporting research results: the Bonferroni vs Holm methods.. Am J Public Health..

[4] CONSORT. The CONSORT Statement.. http://www.consort-statement.org/consort-statement/overview0/.

[5] Jamali R, Pourshams A, Amini S, Deyhim MR, Rezvan H, Malekzadeh R (2008). The upper normal limit of serum alanine aminotransferase in Golestan Province, northeast Iran.. Arch Iran Med..

